# Differentiated instruction for Grade 3 reading challenges: South African teachers in full-service schools

**DOI:** 10.4102/ajod.v14i0.1549

**Published:** 2025-05-16

**Authors:** Thembi A. Phala, Anna J. Hugo

**Affiliations:** 1 Department of Early Childhood Education, College of Education, University of South Africa, Pretoria, South Africa; 2 Department of Language Education, Arts and Culture, College of Education, University of South Africa, Pretoria, South Africa

**Keywords:** full-service schools, Grade 3 learners, different reading materials as content, process and product in the teaching of reading, learning environment, in-service teacher training

## Abstract

**Background:**

We analysed the use of differentiated reading support by Grade 3 teachers and learning support teachers to help learners with barriers to reading in three full-service schools in Tshwane North District in Gauteng Province, South Africa.

**Objectives:**

The study explored how Grade 3 teachers and learning support teachers in full-service schools implement differentiated instruction to support learners with reading difficulties in their classrooms.

**Method:**

A qualitative approach with a case study design was used to collect data from 11 Grade 3 class teachers and 6 learning support teachers in three full-service schools. Data were obtained using semi-structured interviews and classroom observations; thus, an interpretivist paradigm was followed.

**Results:**

The findings showed that different strategies were employed by the participants, but some of the finer applications of differentiated instruction were unused. The following themes emerged: choosing different reading materials as content, the process during the teaching of reading, the product in the teaching of reading, the learning environment and the need for in-service teacher training.

**Conclusion:**

The results highlighted the difficulties inherent in using differentiated instruction based on Grade 3 learners’ reading needs. Many of the learners had specific barriers to reading.

**Contribution:**

The study contributes to the literature on methods of teaching reading in Grade 3 classes. It captures the need for teacher professional development regarding using the finer applications of differentiated instruction in the classroom.

## Introduction

The authors opine that it is a worldwide phenomenon that one of the better changes that occurred over the past 30 years in society and especially in education is the introduction and growth of inclusive education. Inclusive education adheres to the principles that all children irrespective of their abilities or disabilities should be accommodated in schools and their diverse learning needs should be recognised and attended to.

The diversity among learners in classrooms is presented by the different learning needs that learners have. One of the biggest learning needs that young learners have is support in developing their reading abilities. Reading is a fundamental ability for people to achieve success in life. The reason for failure in academic subjects at schools can often be found in inadequate reading abilities (Nel & Nel 2022:121). Keyes et al. (2017:9) aptly state: ‘Reading is the most fundamental skill one must learn while in school. However, difficulty in reading proficiently abounds’. It is thus necessary to attend to the gap in research about approaches to address the inadequate reading abilities of the learners.

Learners who struggle with written text usually read one or more years below their current required grade level without being identified with any learning difficulty (Hall 2009).

According to the 2023 Progress in International Reading Literacy Study (PIRLS), 81% of South African Grade 4 learners are experiencing major challenges when decoding and cannot read with understanding in any language (Mullis et al. 2023). The question arises of whether teachers are ready and trained to support learners with diverse learning barriers, especially reading barriers. We collected data from Grade 3 class teachers and learning support teachers in full-service schools about the methods that they employ to address learners’ reading barriers in their classrooms. Also, we reported on their initiatives to use differentiated instruction to support learners with reading barriers in their classrooms.

## Inclusive education and full-service schools

The main vision of inclusive education is captured well in the 2015 Sustainable Development Goal 4 of UNESCO: ‘Ensure inclusive and equitable education and promote lifelong learning opportunities for all’ (Nel, Nel & Hugo 2022:7). With regard to Africa, Phasha, Mahlo and Dei (2017:3–4) opine that inclusive education should also attend to anti-colonial, de-colonial and integrative views. One of the questions arising from this understanding is how to establish a view of inclusion and social justice in schools that includes the emotional and socio-environmental facets of all learners.

Inclusive education denotes that all children, irrespective of their disabilities, also referred to as impairments, have a right to education in mainstream schools. There are many forms of impairments that could lead to a breakdown in learning. The factors that could be the reasons for a breakdown in the learning process can be classified into two main categories: intrinsic barriers to learning and extrinsic barriers to learning. Intrinsic barriers refer to conditions within a learner, such as blindness, hard of hearing or epilepsy. Extrinsic barriers are conditions outside a learner, such as socioeconomic barriers, the family and school system and a lack of amenities. Reading problems could also be an extrinsic barrier to learning (Nel, Nel & Hugo 2016:10, 21–22.).

The issue of accommodating learners with barriers to learning and development in schools is an issue of concern in South Africa, and policies have been put in place to address it. The Department of Education (DoE) adopted the policy of Inclusive Education in 2001. This policy aims at addressing issues of access and allows schools to be more inclusive in their practices. In response to the policy, some of the mainstream primary schools were converted into full-service schools (FSSs). Department of Basic Education (DBE) (2010:21) reports that FSSs are former mainstream schools that accommodate learners who require low to moderate levels of support. Learners who require low levels of support refer to those learners who need psychosocial support and have visual or auditory processing challenges. Learners who require moderate levels of support have mild hearing or visual impairment, cognitive impairment, epilepsy and cerebral palsy, as well as dyslexia, attention deficit and hyperactivity disorder (Gauteng Department of Education [GDE] 2011:15–16). Learners who have reading barriers fall under this category.

To ensure that FSS teachers are skilled and knowledgeable to support all learners in their classrooms, the GDE has provided these teachers with the opportunity to participate in different programmes (GDE 2010:11). Despite such government intervention, numerous studies conducted in South Africa revealed that learners are struggling to read, including Grade 3 learners (Department of Basic Education 2014b).

Policies in education are constantly changing, and one of the biggest changes that occurred is the growth of inclusive education in many countries, including South Africa. Finkelstein, Sharma and Furlonger (2021:735) claim that five aspects of inclusive education allow teachers to design lessons to undo barriers to learning and teaching for learners who have special needs in education, including an instructional practice, which entails how the teaching and learning processes are organised. This is where differentiated instruction comes in.

## Differentiated instruction

There is a worldwide trend for people to move from urban areas to more densely populated areas, which contributes to making the learner population more diverse. Another tendency is to flee across the borders of countries, which gives rise to an even bigger diversity of learners and their needs. Learners in many schools thus vary greatly in terms of their cultural backgrounds, language competencies, learning styles, motivation and school performance, necessitating teachers to apply differentiation when teaching diverse learners with diverse educational needs (Pozas, Letzel & Schneider 2020). In South Africa, the various home languages of learners, including the home languages of children of legal and especially illegal immigrants, could be added, as well as learners’ competencies in English as a second language, the language of instruction in most schools. Learners thus come from diverse backgrounds with diverse learning and teaching needs.

Tomlinson, the foremost pioneer of Differentiated Instruction (DI), defines it as an inclusive and flexible process that includes the planning, preparation and delivery of instruction to address the diversity of students’ learning needs within the classroom (Tomlinson 2016). Similarly, Coubergs et al. (2017:41) emphasise that DI is not a single teaching strategy but ‘a praxis of teaching a variety of empirically supported strategies across the areas of curriculum planning, assessment and monitoring, instruction, and classroom organisation’. Differentiated Instruction is, therefore, based on the premise that no single teaching strategy can be effective for all learners, especially if the teaching strategy does not fit the learners’ readiness levels, interests and preferred modes of learning (Tomlinson 2016).

Groenewald et al. (2024) did a systematic review and meta-analysis about the success of using DI to improve the learning outcomes of learners in different locations. The research showed major positive impacts of DI on learners’ academic achievements and subject-related abilities. The influence of regional differences was also evident. The outcomes underlined the important role that DI could play in promoting inclusive education.

Additionally, DI has received much attention from different scholars on its effectiveness in improving learners’ reading achievement, especially reading comprehension (Deunk et al. 2015). A study conducted by Magableh and Abdullah (2020) in Jordan about the effect of differentiated instruction on reading comprehension achievement revealed that DI was effective in increasing reading achievement scores in English. Similarly, Reis et al. (2011) studied the effect of DI and enrichment pedagogy on reading achievement in five elementary schools across the United States, whose findings indicated that an enrichment reading approach, with DI and less whole-group instruction, was as effective as or more effective than a traditional whole-group basal approach.

Teachers need to design different activities or differentiate activities according to the learners’ individual learning needs. The role of teachers in a classroom where differentiation is applied, is, therefore, not only to convey the content knowledge but to facilitate the learning process. Thus, they need to differentiate the instructions in such a way that all learners benefit (Heacox 2012:10). In classrooms, teachers can differentiate the following key components in their teaching: content, process, product and learning environment (Heacox 2012:10–11). These components are reiterated by Tomlinson (2017:1), stating that they help to make the teaching situation learner-centred and proactive, leading to curriculum differentiation.

The key components are also captured in the policies of the DBE in South Africa. Content relates to what teachers need to teach, implying what the learners need to learn, know, understand and do (DBE 2011:8). Process entails the strategies, teaching methods, learning support materials, activities engaging in the learning and strengthening of the understanding (DBE 2015:72). Product indicates how learners demonstrate what they are learning or have learned (DBE 2015:72). Learning environment indicates the site where learning is taking place (DBE 2014a:12). This includes both the physical and psychological learning environment.

Ineffective implementation of policies often happens in South Africa. Asriadi et al. (2023) advise that the effective implementation of DI depends on the educational setting in a country. Although DI could be used well in any educational setting, its successful outcomes depend on the influences of the milieu.

## Research methods and design

A qualitative research approach with a case study design was used in this study. According to Bertram and Christiansen (2016:26), a case study design could provide an understanding of how people understand the contexts within which they live and work. The case study design helped the authors to look at the chosen schools within which the teachers performed their work in a bounded setting. Purposeful sampling was used to ensure that all the participants were well-trained teachers with years of teaching experience. In this study, three FSSs from the same district were included.

Grade 3 class teachers and learning support teachers in three FSSs in Tshwane South District, Gauteng Province, South Africa, were suggested by the GDE’s chief education specialist to participate in the study. These schools were referred to as Schools A, B and C. All schools had basic infrastructure and classroom furniture. Sepedi and isiZulu, two of the official languages in South Africa, were used as the languages of learning and teaching, except in School C, where English was used. The participants were selected based on their experience in teaching Grade 3 learners, training received to support learners with reading problems using differentiation instruction and their availability and willingness to participate in the study. Seventeen Grade 3 teachers, comprising 6 learner support teachers and 11 Grade 3 class teachers, were selected.

An additional Grade 3 teacher from the neighbouring FSS was also involved during the pilot study. A pilot study was conducted before the actual research took place to test the research questions and identify questions that could be unclear. This resulted in the revision of some questions. In this study, LST refers to a learner support teacher, and GR3CT refers to a Grade 3 class teacher. All participants were females and professionally trained as teachers. The participants were, however, different in terms of their home language, qualifications and years of teaching experience in teaching Grade 3 in a FSS.

Trustworthiness must be considered to assess the quality and the value of a research project using qualitative data. We considered the four criteria, as described by Lincoln and Guba, to establish whether the findings of the study reflected the aims of the study: credibility, dependability, confirmability and transferability (Alexander 2019).

### Data collection method

The data for the study was gathered in Grade 3 reading classes through semi-structured interviews and classroom observations. Semi-structured interviews were scheduled with the participants for an hour for 3 weeks after normal teaching hours. The semi-structured interviews comprised open-ended questions. The open-ended questions were designed based on a literature review about reading and DI that we conducted. The comments on the questions of a knowledgeable colleague were also attended to. This type of interview allowed the participants to express their personal views and understanding of how they, as GR3CTs and LSTs in FSSs, used DI to support learners with barriers to reading.

In addition to the semi-structured interviews, classroom observations were conducted depending on the availability of participants and the school periods when reading classes are offered. We carefully worked through the classroom observations to identify themes. The themes arising from the classroom observations were triangulated with the themes from the semi-structured interviews. We avoided potential biases of participants by randomly selecting the names of possible participants provided by the district official. All the data were kept in a locked cupboard in a locked office.

This study was framed by Vygotsky’s socio-cultural theory. This theory describes the concept of the Zone of Proximal Development (ZPD), mediation and cultural tools. The ZPD defines the distance between a learner’s current level of development and the potential level of development that can be attained through mediation and the support of able adults or peers (Shabani et al. 2010:237–238). Thus, a teacher can ensure that effective teaching takes place by using, for instance, DI in the classroom so that the level of reading development of a learner or a group of learners could be considered in the planning of reading.

### Data analysis

Data analysis is a technique used to structure, bring order and give meaning to data collected (ed. De Vos 2015:397). For this study, a thematic content analysis was used to analyse data. Before we analysed the data, we read and reread it to identify the units of meaning and cluster them. From the analysis, five themes about differentiated instruction to teach reading were identified. We discussed the themes in the findings section.

### Ethical considerations

Ethical clearance to conduct this study was obtained from the University of South Africa College of Education Ethics Review Committee (No. 2017/05/17/30112508/17/MC). To ensure confidentiality, the names of participants were not used at all. Written consent was obtained from the GDE and the principals of the schools. The LSTs and GR3CTs who acted as participants also completed written consent forms.

### Results

The study explored how Grade 3 teachers and learner support teachers in three FFSs used differentiated instruction when teaching reading and supporting learners with barriers to reading. The principal finding was that the participating teachers understood most of the principles of differentiated instruction to support learners with reading problems. There were, however, some of the finer applications of DI that they could have used. This article reports on five themes about DI emanating from the study, including the need for teachers for specific training in DI (see Table 1).

**TABLE 1:** Summary of themes.

Theme 1	Choosing different reading materials as content when planning reading lessons
Theme 2	The process during the teaching of reading to Grade 3 learners
Theme 3	The product in the teaching of reading to Grade 3 learners
Theme 4	The learning environment
Theme 5	The need for in-service teacher training regarding differentiated instruction

#### Theme 1: Choosing different reading materials as content when planning reading lessons

It could be difficult for teachers to plan various lessons according to the abilities and needs of different learners because teachers also must adhere to the curriculum requirements and content of the Department of Education.

It was clear that the participants understood that they had to differentiate the choice of reading materials for their Grade 3 learners who were having problems reading Grade 3-level texts. Most of the participants grouped their learners according to their reading levels, and they supplied reading materials according to the reading levels of the learners, thus taking the levels of reading development of the learners into consideration. This group of participants also applied the educational principle to move from the known, which is what the learners could read, to more difficult sections and words unknown to the learners.

One of the participants said that she usually used a ‘lower book’ for learners who could not read. She would use books like ‘Sounds like fun’ because these books have basic words consisting of three letters (GR3CT9, female, primary school teacher). Another participant used curriculum differentiation because, in each group, there were learners who were at the same level. They would read a paragraph containing words with an ‘a’ sound. If perhaps they planned to read a paragraph with most words with an ‘a’ sound, she would write the words in the learners’ home language for those learners who could not read. Examples were ‘apeya’ (cook) and ‘apolo’ (apple), and she would also have pictures to explain the words. For those who could read, she would give them a paragraph to read because they understood what she was saying. If these learners had to point to a word such as ‘akanya’ (think), they could do it (LST2, female, primary school teacher).

Participant LST4 mentioned the use of different books based on the reading levels of the learners, stating that she sometimes used different books to cater for different learners. She gave those learners extra books so that they could read at home with their parents assisting them (LST4, female, primary school teacher). LST6 made provision for a learner’s reading interests. She explained that she planned the reading support using pictures and simple storybooks. She would then give a learner an opportunity to choose what he or she wanted to read as the learners’ interests differed. Thus, variety in the reading material was provided (LST6, female, primary school teacher).

During the observations, it was noticed that in all classes, the teachers pasted different charts, for example, alphabet charts, phonic charts and number charts, on the walls. The alphabet-pasted charts had capital and small letters of the alphabet. This assisted learners in knowing and recognising all the letters and knowing how to write each letter. The phonic charts consisted of the first letter of a word, such as ‘a’ with a picture of an apple or ‘b’ with a picture of a boy. The use of these phonic charts was problematic in two of the schools because the learners’ home languages as the languages of teaching and learning were not English. One of the LSTs had, however, created her own animal chart. There were pictures aligned with the topic that she was teaching. This teacher pasted a chart illustrating different animals on the wall, such as ‘tlou’ (elephant) and ‘kwena’ (crocodile). The teacher also used the picture of the ‘kwena’ to emphasise the sound ‘kw’ that she was teaching (LST1, female, primary school teacher).

#### Theme 2: The process during the teaching of reading to the Grade 3 learners

The study revealed that all the teacher participants followed the mixed ability grouping when they taught reading. They differed, however, in the way they implemented the concept and in the way it was used. The following excerpts serve as examples.

Participant GRTCT2 downgraded the lesson by moving down a grade. Instead of using Grade 3 materials, she would use Grade 2 materials. There was no use for her as a teacher to move forward when some learners were still struggling with a certain letter. By differentiating the content in a lesson, struggling learners were able to read, leaving out emphasis on capital letters and punctuation (GRTCT2, female, primary school teacher). Because this participant helped her learners to be successful when they read, this could serve as an important way to motivate the learners who have reading problems to read.

Participant LST6 took it further when stating that she normally started with the pictures. Then, the learners got a chance to say anything that they thought the pictures were about. Afterwards, LST6 read the story. Then, she placed the words next to the pictures and read the words. The learners then read after her. Next, she worked with the learners in groups (LST6, female, primary school teacher).

The participants who used grouping in the study made use of the intentional composition of learners working or actually reading groups (Pozas et al. 2020). The authors opine that the organised heterogeneous groups should be based on reading performance of the learners. One basic lesson in which different activities are built in could be a solution. Regarding differentiating reading activities, one participant, an LST, used one lesson for the class, but she differentiated the activities (LST1, female, primary school teacher).

Participant LST2 used differentiation to help learners who were at Grade 3 level and had problems with the sounds of their home language. She planned the lessons for the learners’ level of needs. For those learners who struggled a lot, she put more effort into their lessons by helping them to differentiate the different sounds or to learn sounds (LST2, female, primary school teacher).

Participant LST3 said that not all the lessons could be one-size-fits-all. She had to plan well, as learners’ potential was not the same. Learners did not grasp new or unknown language at the same level. Curriculum differentiation allowed her to plan according to the learners’ abilities. She avoided it to give the slow learners more activities because their concentration span was short and too many activities would only frustrate them. Working with the second group, she gave them the little work that she had done with the first group, adding a little more work to them until this group was at the same level as the last group. When the last group was familiar with the new texts, they were ready to go to their class, as they did not need the support of a learning support teacher anymore (LST3, female, primary school teacher).

Another participant who was a Grade 3 classroom teacher explained that when she planned for reading, she also planned for other things:

‘You know, when I plan reading, I plan reading as I plan for other things. She remembered that her learners were not the same. Sometimes she realised that when some learners were busy with three letters, others were busy with one letter, and others were dealing with two letters. So, when she planned her lesson, she used one lesson but with different parts. In this way, one lesson catered for different learners and their different learning needs.’ (GR3CT3, female, primary school teacher)

The same teacher explained that when, for instance, she used the diphthong ‘thwa’ (in the mother tongue Sepedi of the learners) in sentences to her class, she would at first only teach just the letter ‘t’ in words or in sentences to the learners with reading problems.

#### Theme 3: The product in the teaching of reading to Grade 3 learners

It is explained in the Screening, Identification, Assessment and Support (SIAS) policy, which is a manual for teachers of the DBE. The product indicates ways by which the learners will communicate learning and understanding. This means that during baseline assessment or informal assessment, learners will demonstrate what they are learning or have learnt (DBE 2015:72). Thus, specific reading lessons according to the reading needs based on the assessment of specific learners or a learner could be planned so that differentiated instruction is applied.

Participant LST5 explained that before she did the planning, she went to the classrooms to check the programmes class teachers were doing before they sent learners with reading problems to her. If she realised that a learner or group of learners knew certain letters and sounds, she would then start with blends. Then, they would take a reading programme or story concentrating on blends. Afterwards, the learner or learners would read after she had read. This would help her to see if learners were still struggling or whether they could be improving (LST5, female, primary school teacher).

Participant LST3 ensured that the learners that she helped knew all the letters and sounds of the alphabet, especially the vowels and the role of vowels in words. She would leave out a vowel in a word to assess if learners know for instance the vowel ‘a’. Instead of writing ‘katse’ (cat) she would write ‘k…tse’. By doing this she could check if the learners realised that something was missing, and the word is not a ‘katse’ anymore without the letter a (LST3, female, primary school teacher).

#### Theme 4: The learning environment

The site and thus the learning environment where instruction and, specifically reading instruction, takes place must be considered as well. This entails both the physical and psychological learning environment. The learning environment could play an important role in the planning and success of DI to enhance the teaching of reading. The learning environment of a classroom entails the ‘climate’ or the ‘tone’ of a classroom, which includes things like the arrangement of the furniture, the lighting and whether the classroom is conducive to teaching and learning to take place.

During observation, we noticed each LST supported a group of 12 learners. The learners were seated in groups of three in five LST classes. In LTS3’s class, the learners were seated in rows. Each group of learners were supported once a day. All the classes were print-rich, with different charts pasted on the wall. There was also a reading corner in all the classes to allow for independent reading, and each learner had a reading book (Phala 2019:172–176).

In Grade 3 classes, the seating arrangement of learners was different. In all 11 classes, the learners were seated in groups with two desks facing each other to accommodate a larger group. The number of learners in these classes ranged from 32 learners to 49 learners. All classes had a mobile library used as a reading corner. During the reading support, all teachers used a big book to read the story from, while the learners used the photocopied story to read. The researchers noticed that there were readers in the mobile libraries, but the learners were not provided with these readers during a reading lesson (Phala 2019:172–176).

Peer teaching has been used by many teachers because learners usually normally understand each other well. In the study, peer teaching was also mentioned. One learning support teacher (LST4) said:

‘In class, we sometimes make a competition where learners have to read with fellow learners. If that learner is not doing well, the peer will also advise and read in front of that learner so that the learner can be motivated. Or sometimes they help them to read together, and then that is how I do my support in class.’ (LST4, female, primary school teacher)

#### Theme 5: The need for in-service teacher training regarding differentiated instruction

The introduction of FSSs in the South African school system meant that teachers, LSTs and the management of schools must be trained to understand what FSSs entail. The need for in-service teacher training was stated in 2008 in the National Policy Framework for Teacher Education and Development (NPFTED) (DoE 2008:3). The main aim of the policy is to improve teachers’ professional competency so that they would be able to address learners’ specific needs to help them to better their performance at school (DoE 2008:7–8). The need for in-service training by the FSS teacher participants arose from the study.

A Grade 3 classroom teacher (GR3CT4) said that they were always in need of support in the form of in-service training because there were always those learners who experienced different problems every year. She needed support from the district on how to deal with learners with severe writing and reading difficulties, thus:

‘With reading, I have tried so many methods, so I need training through workshops on how to make these learners understand phonics. Now, in our workshops, they only tell us the things that we know, not something new.’ (GR3CT4, female, primary school teacher)

It was evident that this teacher had difficulties in knowing how to differentiate her teaching when dealing with learners with serious writing and reading problems.

This was reiterated by another GR3CT (GR3CT5, female, primary school teacher), who opined that training in the Learner Support programmes could be useful, especially on different methods to be used when dealing with reading problems. She said that classroom teachers wanted to attend the workshops for LSEN (Learners with Special Education Needs – a term previously used in policies and training) programmes which were offered for the learning support teachers. She felt that all teaching staff members at full-service schools should be trained on how to deal with reading problems. She asked for a lengthy workshop, maybe even for a week.

It was clear that most of the participants in the study wanted to improve their knowledge about the teaching of reading with the aim of providing more and better support for their learners with reading problems.

It is a given that society is changing and evolving. This necessitates in-service teacher training to ensure that teachers are kept abreast of changes regarding subject content and pedagogical knowledge to meet the changing demands of the 21st century (Kyeremeh, Adzifome & Amoah 2022:67). The use of DI as a key element of teaching and learning in an inclusive classroom could be new to many teachers who have been teaching for many years. It is thus advisable that the knowledge that teachers and LSTs in FSSs have about DI be investigated and that the in-service training that they receive, should address the gaps in their knowledge about DI. This could help them differentiate the curriculum in FSS classrooms especially when they teach reading to young learners.

John and Segalo (2021:959) state that this lack of collegiality results in the climate of the school not being enabling or friendly. This could happen in FSSs because LSTs are better qualified than classroom teachers. If the principal and the management of FSSs could address a lack of collegiality at their schools, the LSTs could train the classroom teachers to address learners’ learning and reading problems using their knowledge and additional training backgrounds. This could address the need for in-service teacher training to a certain extent. Additional in-service teacher training should be done by the various departments of education.

## Discussion

When teaching reading, the use of well-selected reading materials is important. This is even more important when teaching Grade 3 learners who have different reading abilities and are reading at different reading levels. In the study, most of the respondents used different reading materials based on the learners’ reading levels. There were, however, not enough different interesting and age-appropriate reading materials available in all the classrooms. Sun (2023) states that in extensive reading programmes, various reading materials, including easy materials on several topics, should be available. It is important that readers choose their own materials and read them for pleasure. Teachers should also act as role models for readers.

Learners who have reading problems could benefit when well-planned and applicable resources are used during reading lessons and, if possible, at home. Teachers could scaffold learners’ reading, either in words by explaining or by giving examples. The topic could also be explained with the help of visual materials. Lynch (2020) suggests that charts, drawings, diagrams and reference guides could be used. If available, technology could also be used in the form of a video clip.

In the study, it became clear that it was the LSTs who acted as participants who indicated that they used additional texts and reading materials aligning with learners’ reading levels as part of curriculum differentiation to support their learners with reading problems. It is a pity that the readers in the mobile libraries were not used by the classroom teachers to support learners. Just like the use of reading materials, the use of additional resources such as charts and pictures should be well-planned. Using additional materials that are inappropriate, serves no objective.

During the process of teaching of reading, differentiation of activities can have potential to supply support for learners with reading problems and other learning activities. This is reflected well in the data obtained from the participants, as some of them clearly planned different activities, sometimes using the same content. The differentiation must be planned and scaffolded by the teacher. Various activities should be planned based on the different learners’ reading abilities. This would, however, necessitate the constant assessment of learners’ progress in reading.

There could be pitfalls when learners are grouped into various reading groups and teachers should be aware of them. Pitfalls include the problem that classmates could ridicule young learners if they are in a group where weak learners are placed. If learners are ‘down-graded’ as mentioned by the participants, the interest level of the texts could not be age-appropriate. Professional literature clearly shows that flexible grouping should be used. Teachers should thus use a variety of grouping patterns. Although there is no right or wrong method to organise learners in reading groups, the grouping should be flexible and be changed regularly. Learners’ interests and skills should be considered and regrouping should take place (Weisskopf 2009:138).

There was no evidence in the study that the participants were aware of possible pitfalls that could arise if learners were placed in different reading groups. Neither the classroom teachers nor the learning support teachers gave any indication that they used a variety of grouping patterns or that their groupings were flexible.

As far as the product of the teaching of reading is concerned, differentiation of activities could also involve the assessment of learners’ reading and their reading comprehension. After a story or text has been read by all the learners, the assessment of their comprehension can be done in different ways. Learners could complete questions about key ideas and events in the story. Learners with reading problems can draw a story on the web (Lynch 2020). Learners could also be scaffolded by letting them fill in words only. A graphic organiser of the text to be read could also be provided. A teacher should consider what type of reading an assessment task will require and how much time the assessment will require for a learner with a reading problem to read the task.

Weselby (2021) refers to the use of flexible grouping in their teaching of reading, based on the teachers’ assessment of learners’ knowledge of phonics, for instance, or on the learners’ interests in certain books. Suppose the participants in this study, as well as other teachers and LSTs in FSSs, are trained to use grouping as part of differentiated reading instruction and reading assessment. In that case, they should, however, also be informed about the pitfalls that could arise.

During the collection of data, the researchers did not have any clear plan for the assessment of learners’ reading at the sites that were visited. Lynch (2020) draws attention to the important factor in the assessment of reading, which is that the assessment of the reading should also be planned. For additional reading instruction to be successful, the specific reading needs of learners must be attended to. This requires the planned and continuous assessment of the reading levels of individual learners. After the assessment, learners could be grouped, and the reading instruction of each group planned accordingly.

During the study, the researchers noticed two aspects of the learning environment which contributed to the enhancement of DI at the schools where the research was conducted. One aspect was the fact that LSTs who could provide additional remedial instruction to smaller groups of learners or to individual learners were introduced at the schools. The use of LSTs is one of the pillars of FSSs, and at the three study sites, the LSTs were providing support to many learners both individually and in small groups.

The other aspect was peer teaching, when well used and planned, could have a positive psychological effect on certain learners’ reading progress. It was encouraging that some of the participants were using peer teaching in an attempt to support learners with reading problems in their classrooms. This aligns with international research by Gubalani et al. (2023), who state that ‘supporting the notion that peer tutoring contributed to the improvement of reading comprehension’. Fuchs and Fuchs (2005) indicated that peer teaching has the potential to develop young learners’ literacy abilities, including learners with reading problems. These days, many experts, including UNESCO, argue that peer teaching is used successfully in many countries. Peer teaching could help learners make academic progress, such as in their literacy abilities, but it could also enhance their self-esteem and attitude toward schoolwork and the school in general. The activities during peer teaching when reading should be well structured (Blanch et al. 2012:1685).

The authors are aware that a lack of collegiality sometimes exists in schools where certain teachers are better trained than others. John and Segalo (2021:959) state that there are schools in South Africa where a lack of collegiality results in the climate at the schools not being enabling or friendly. This could happen in FSSs because LSTs are better qualified than classroom teachers. If the principal and the management of FSSs could address a lack of collegiality at their schools, the LSTs could train the classroom teachers to address learners’ learning and reading problems using their knowledge and additional training backgrounds. This could address the need for in-service teacher training to a certain extent. Additional in-service teacher training should be done by the various departments of education.

During in-service teacher training, the importance of DI to provide support to learners with different and individual learning needs should be stressed.

## Recommendations and implications

The research findings arising from this study showed that knowledge about inclusive education is not enough for successful teaching in FSSs. This is important during Grade 3 reading lessons, where learners receive their final formal instruction in reading, preparing them for Grade 4 and onwards, where they must be able to read in order to learn. If Grade 3 learners do not manage to read fluently and with comprehension, this could have dire implications for their success at school. As discussed earlier, the PIRLS results of 2023 revealed that the majority of Grade 4 learners in South Africa could not read with understanding.

It is recommended that all primary school teachers who teach reading should receive in-service teacher training to identify learners’ reading problems and reading needs. These teachers should also be retrained where necessary to use different reading methods and strategies to suit the learning needs and learning abilities of their learners. All learners who require moderate levels of support because of a mild barrier to learning and development, including reading problems, should be assisted in FSSs by specifically trained reading teachers. It is vital that learners’ specific reading problems should be attended to.

It is clear that to have a policy about FSSs is not enough and that practical application of the policy in the classroom should be considered by the various departments of education. Ineffective implementation of policies often happens in South Africa. Asriadi et al. (2023) share that it is not the policy but the educational milieu that will lead to the successful implementation of DI in a country. It is recommended that teachers in FSSs receive continuous professional training, especially regarding the teaching of reading and how DI could be used to support learners with reading problems.

The teachers in FSSs should be well acquainted with the use of different reading methods, and thus, they should be flexible to use various methods and strategies according to the reading abilities and needs of individual learners. Special attention should be given to the choice of reading content based on learners’ age and interests, as well as the learning environment in the classroom. The environment in the classroom must stimulate reading curiosity and interest in reading by including interesting reading materials and additional resources such as pictures, charts and word walls. If possible, FSSs should have libraries to enhance reading among learners further. The provision of additional reading materials and books in libraries should be included in the budgets of education and FSS departments. Thus, DI could become a key element in the reading class in FSSs.

## Limitations

The study was conducted in Tshwane South District of Gauteng. The results thus reflected the situation regarding the use of differentiation during reading instruction in three FSSs in a specific area in Gauteng. As such, the results only reflected the use of differentiation during reading lessons in the classrooms of a group of teachers in one province of South Africa. Not all the GR3CTs and LSTs in the three FSSs were selected as participants; thus, the results reflected the teaching of reading of the 17 participants. The study was also carried out in the schools of black children, and other FSSs not in this category were left out. The findings may not be representative of other FSSs in other parts of South Africa.

## Conclusion

From the data, the teachers in the FSSs, who were the participants in the study, were informed about most of the principles of inclusive education. The application of the principles of differentiated instruction to support learners with barriers to reading were, however, not always achieved. Although the barriers to learning and development that some of the learners in the three FSSs in the study were low to moderate, not all of these learners could be helped and supported to overcome their barriers to learning and development, especially to reading.
